# Wound Dressing: Combination of Acacia Gum/PVP/Cyclic Dextrin in Bioadhesive Patches Loaded with Grape Seed Extract

**DOI:** 10.3390/pharmaceutics14030485

**Published:** 2022-02-22

**Authors:** Cinzia Pagano, Francesca Luzi, Maurizio Ricci, Alessandro Di Michele, Debora Puglia, Maria Rachele Ceccarini, Tommaso Beccari, Francesca Blasi, Lina Cossignani, Aurélie Schoubben, Sara Primavilla, César Antonio Viseras Iborra, Luana Perioli

**Affiliations:** 1 Department of Pharmaceutical Sciences, University of Perugia, 06123 Perugia, Italy; maurizio.ricci@unipg.it (M.R.); mariarachele.ceccarini@unipg.it (M.R.C.); tommaso.beccari@unipg.it (T.B.); francesca.blasi@unipg.it (F.B.); lina.cossignani@unipg.it (L.C.); aurelie.schoubben@unipg.it (A.S.); luana.perioli@unipg.it (L.P.); 2 Department of Materials, Environmental Sciences and Urban Planning (SIMAU), 60131 Ancona, Italy; f.luzi@staff.univpm.it; 3 Department of Physics and Geology, University of Perugia, 06123 Perugia, Italy; 4 Civil and Environmental Engineering Department, University of Perugia, UdR INSTM, 05100 Terni, Italy; debora.puglia@unipg.it; 5 Istituto Zooprofilattico dell’Umbria e delle Marche “Togo Rosati”, 06126 Perugia, Italy; s.primavilla@izsum.it; 6 Department of Pharmacy and Pharmaceutical Technology, Faculty of Pharmacy, University of Granada, 18071 Granada, Spain

**Keywords:** acacia gum, wound, grape seed extract, patch, bioadhesion

## Abstract

The success of wound treatment is conditioned by the combination of both suitable active ingredients and formulation. Grape seed extract (GSE), a waste by-product obtained by grape processing, is a natural source rich in many phenolic compounds responsible for antioxidant, anti-inflammatory, and antimicrobial activities and for this reason useful to be used in a wound care product. Bioadhesive polymeric patches have been realized by combining acacia gum (AG) and polyvinylpyrrolidone (PVP). Prototypes were prepared by considering different AG/PVP ratios and the most suitable in terms of mechanical and bioadhesion properties resulted in the 9.5/1.0 ratio. This patch was loaded with GSE combined with cyclic dextrin (CD) to obtain the molecular dispersion of the active ingredient in the dried formulation. The loaded patch resulted mechanically resistant and able to release GSE by a sustained mechanism reaching concentrations able to stimulate keratinocytes’ growth, to exert both antibacterial and antioxidant activities.

## 1. Introduction

The research in the wound dressing field is growing due to the necessity of having products efficacious in wound healing and, at the same time, accepted by the patient.

Many wound dressings available on the market are designed to adhere to the skin by means of adhesives, and this solution represents a problem since the removal is painful and responsible for the damage of the surrounding newly formed tissue.

Biodegradable formulations obtained avoiding the use of adhesives represent a possible solution as reported by other authors [1,2,3,4].

Hydrophilic polymers, such as polysaccharides [5], could be considered suitable for this application. In literature, it is described the preparation of films based on polysaccharides such as agarose [6], alginate [7], that coming from marine algae [8], and cellulose [9].

Currently, medications based, for example, on alginates (e.g., Algisite^®^ Ag), hyaluronic acid (e.g., Hyalo4^®^), cellulose and cellulose derivatives (e.g., Aquacel™) can be found on the market.

An interesting material useful for wound medication is represented by the biopolymer acacia gum (AG), a dry exudate of Sub-Saharan acacia tree (Acacia Senegal and Acacia Seyal). AG is a water-soluble polysaccharide with high molecular weight (400–1000 kDa), widely used in cosmetic products for the treatment of skin irritations, burns, and superficial excoriations [10]. It is a safe excipient, classified as generally recognized as safe (GRAS) by FDA, largely used both in pharmaceutical and food industries [11]. AG is also applied as a thickening and gelling agent in both cosmetic and pharmaceutical products, as well as to fix the flavoring substances in the food industry [12]. AG is a cheap raw material, completely biodegradable, with a high thickening capacity at different pH values and temperatures [13]. Moreover, the antioxidant activity was observed as well, mainly due to the inhibition of the lipid peroxidation superoxide radical formation [14].

In recent studies, AG was successfully applied in the development of dressing for the topical delivery of antibiotics, anesthetics [13,15,16].

The use of AG alone does not allow to obtain a formulation with suitable mechanical properties necessary to ensure resistance to mechanical solicitations as removal from the packaging, application on skin, adaptation to every surface. Thus, the combination with another polymer is mandatory. In literature, it is reported that AG can be combined with Carbopol [15], PVA [17], alginate [16], and PCL [18].

In this study polymeric patches, prepared combining AG with polyvinylpyrrolidone (PVP), were considered and characterized. PVP is a polymer largely used to improve film mechanical properties [19] and, for this reason, here is considered as a suitable candidate for the improvement of the weak mechanical performance of AG. The choice of the most suitable active ingredient must be considered for the success of therapy. The developed patches were loaded with a grape seed extract (GSE), known for its antioxidant, anti-inflammatory, and healing properties [20]. GSE has already been used in commercially available products (solutions) intended for wounds treatment such as NutriBiotic^®^, Citricidal™ [21]. However, the solutions show a limited residence time and are not able to protect the damaged area. To overcome these problems GSE has been formulated for example as nanoparticles [22] and aerogel [23]. GSE was also loaded in films obtained by electrospinning [24] or by casting method [25] used both in the food field and wound treatment [26,27]. Formulations in which GSE has been combined with AG/PVP blend cannot be found in the literature. This research is focused on the realization of AG/PVP patches loaded with GSE and on the evaluation of the activity of the extract released from the developed formulation (cytotoxicity, healing, antibacterial) as well as its influence on patch mechanical properties were investigated.

## 2. Materials and Methods

### 2.1. Materials

Acacia gum, ethanol 96% (EtOH), Folin–Ciocalteu, gallic acid, ABTS radical cation, TPTZ(2,4,6-Tri(2-pyridyl)-s-triazine),(±)6-hydroxy-2,5,7,8-tetramethylchromane-2-carboxylic acid standard (Trolox, 97%), 2,2-diphenyl-1-picrylhydrazyl (DPPH), hydrochloric acid (HCl), ferric chloride (FeCl_3_), sodium acetate (NaOAc), sodium carbonate (Na_2_CO_3_), acetic acid (AcOH) and gallic acid (GA) were purchased from Sigma Aldrich (Milan, Italy). Glycerol and polyvinylpyrrolidone (PVPK30, average MW 40,000 Dalton) were supplied by A.C.E.F. s.p.a., (Fiorenzuola d’Arda, Italy). Grape seed extract (GSE, Leucoselect Indena) was purchased from Farmalabor (Canosa di Puglia, Italy). Cyclic dextrin (CD) was a gift from Alikapharma (Gorla Minore, Italy). Calcium chloride was from Carlo Erba Reagents S.r.l. (Milan, Italy).

Ultrapure water was obtained by reverse osmosis process in a MilliQ system Millipore (Rome, Italy). Other reagents and solvents were of analytical grade and used without further purification.

The simulated wound fluid (SWF) pH 6.5 was prepared by dissolving 8.30 g of NaCl and 0.28 g of CaCl_2_ in 1000 mL of ultrapure water.

For microbiological studies test media were prepared as follows:

- agar-well diffusion test medium: deionized water (containing agar 13%), meat extract (3%), sodium chloride (10%), glucose (4%), dibasic potassium phosphate (1%) and meat peptone (5%); after preparation the test medium was autoclaved.
- Brain Heart Infusion (BHI) Broth; deionized water, BHI (3.7%, Biolife Italiana Srl, Milan, Italy).
- Mueller Hinton Broth with 5% Blood; deionized water, Mueller Hinton Broth (2.2%, Biolife Italiana Srl, Milan, Italy), Horse Lysate Blood (5%, Allevamenti Blood di Fiastra Maddalena, Teramo, Italy).
- 5% Sheep Blood Agar; deionized water, Columbia Agar Base (4.4%, Microbiol Srl, Macchiareddu, Cagliari, Italy), Defibrinated Sheep Blood (5%, Allevamenti Blood di Fiastra Maddalena). Bacterial suspension at concentrations of 1 × 10^5^ CFU/mL was used for the antimicrobial test.

### 2.2. Methods

#### 2.2.1. Patches Preparation

Four kinds of patches (having a different AG/PVP ratio) were prepared by solvent casting method [28] starting from hydrogels whose compositions are reported in Table 1. Unloaded patches were prepared starting from glycerol-water solution to which PVP was added under magnetic stirring (600 rpm) at room temperature (R.T.). Finally, AG was added and mixed up to obtain complete hydration of both polymeric components. In the case of loaded patches, the CD was solubilized in the water-glycerol mixture to which GSE was then added (GSE content in the final patch was 1.23 mg/cm^2^). PVP was introduced to the obtained solution, and, after gel formation, AG was added.

**Scheme 1:**
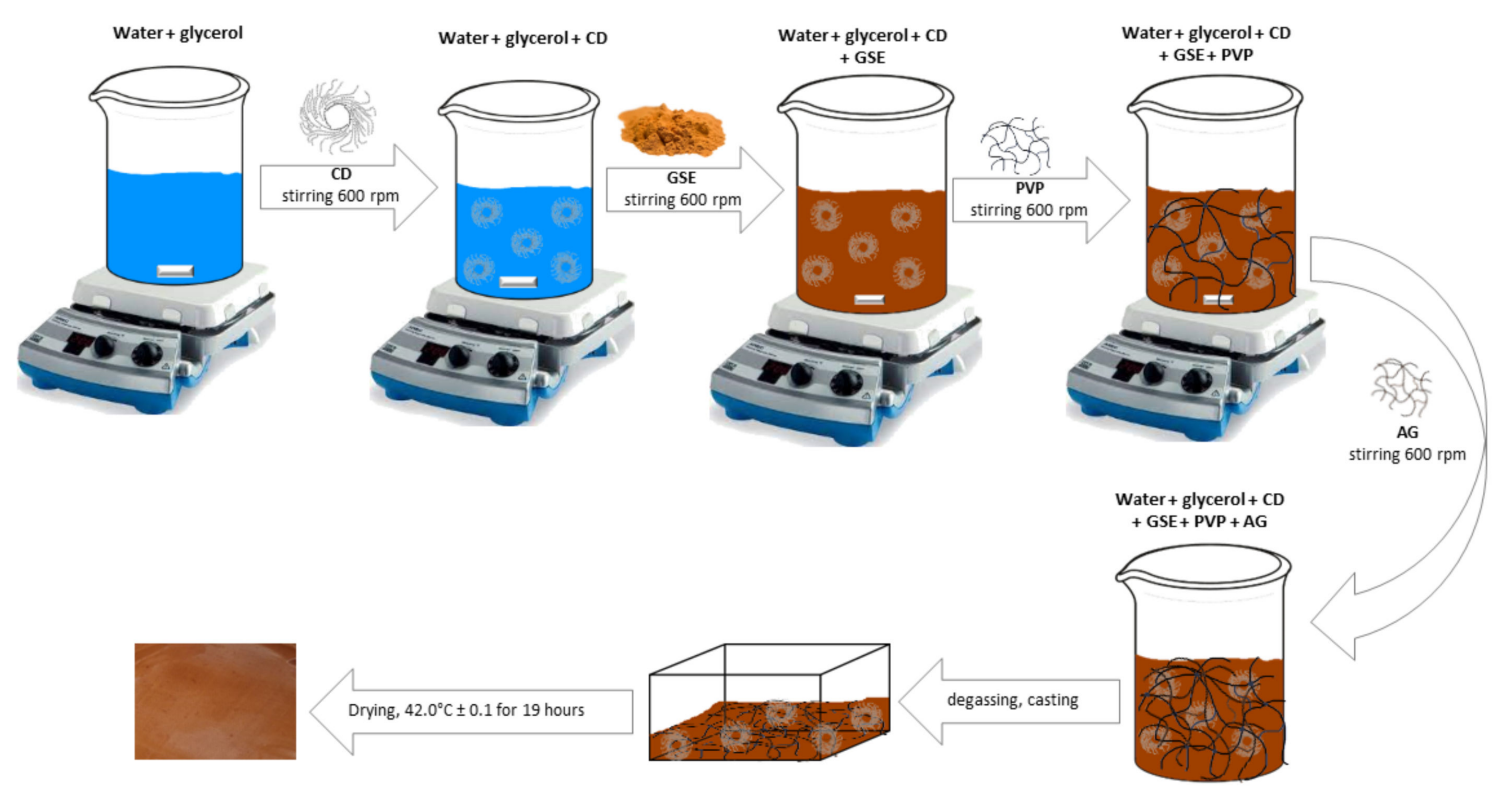
Schematic representation of patch preparation.

The air incorporated during the mixing was removed from hydrogels by using a THINKY ARE-250 mixer (Thinky Corporation, Tokyo, Japan) working at 2000 rpm, for 5 min R.T. Afterwards, 70 g of the prepared mixtures were casted into rectangular Teflon molds (10.5 cm × 15.5 cm), then placed in a ventilated oven at 42.0 °C ± 0.1 for 19 h (Scheme 1).

#### 2.2.2. Thermogravimetric Analysis

Thermogravimetric and DTG analyses of the raw AG and PVP and the prepared patches were performed by using a TG/DTA Seiko 6300 (Seiko Instruments, Torrance, CA, USA).

Each patch was cut in similar portions (weight 10 mg) and placed inside small alumina crucibles. The instrument measures the weight variation of the sample during isothermal or dynamic heating conditions. Tests were performed by heating the samples between 30 and 900 °C at 10 °C/min, nitrogen flow at 250 mL/min.

#### 2.2.3. Total Phenolic Content and Antioxidant Activity

The total phenol content (TPC) of the patches solubilized in water was measured by Folin-Ciocalteu assay [29]. The solutions were diluted with water and then 1 mL of this aqueous solution was mixed with Folin-Ciocalteu reagent and 20% Na_2_CO_3_ solution. After 90 min at R.T. in the dark, the absorbance was measured at 750 nm (ʎ_max_) by using a Lambda 20 UV–Vis spectrophotometer (PerkinElmer, Waltham, MA, USA). A calibration curve in the concentration range of 0.012–0.075 mg/mL was prepared using gallic acid, and the results were expressed as mg of gallic acid equivalents (GAE) per patch having a surface of 1 cm^2^ (mg GAE/cm^2^ patch). The determination was carried out in duplicate.

The radical scavenging activity of the patches was measured by ABTS assay. ABTS^•+^ was obtained by preparing a mixture as described by Urbani et al. [30]. The mixture was left in the dark at R.T. overnight and then diluted with H_2_O until the absorbance was about 0.70 at 734 nm (ʎ_max_). After the addition of 4 mL of diluted ABTS^•+^ solution to 60 μL of the patch hydroalcoholic solution, the reaction was left in the dark at R.T. for 6 min. After that, the absorbance was measured at 734 nm (ʎ_max_). A calibration curve was prepared using Trolox standard solutions and ABTS values were expressed as mg TE/cm^2^ patch. The antioxidant activity of the patches was measured by DPPH assay following the method described by Pollini et al. [31]. DPPH methanolic solution was added to the patch solution. After 30 min in the dark, the absorbance was measured at 515 nm (ʎ_max_). A calibration curve was prepared using Trolox standard solutions and ABTS values were expressed as mg TE/cm^2^ patch.

The reducing power of the patches was determined by FRAP (ferric reducing antioxidant power) assay. The FRAP working reagent consisted of 300 mM acetate buffer (pH 3.6), 10 mM TPTZ in HCl 40 mM and 20 mM FeCl_3_, considering a ratio of 10:1:1 (*v*/*v*/*v*). An aqueous solution of both patches was diluted with 900 μL of distilled water and then FRAP reagent (2 mL) was added. After 30 min at R.T. in the dark, the absorbance was measured at 593 nm (ʎ_max_). A calibration curve was prepared using Trolox standard solutions and FRAP values were expressed as mg Trolox Equivalents (TE) per patch having a surface of 1 cm^2^ (mg TE/cm^2^) [31]. All determinations (ABTS, DPPH, and FRAP assays) were carried out in duplicate.

#### 2.2.4. Mechanical Characterization

Tensile tests were performed by using a digital microprocessor instrument (Llyod LR30K, Lloyd Instrument, Fareham, UK). The patches were cut in portions 100 mm × 10 mm (UNI ISO 527) to have a useful length of 50 mm. The experiment was performed at 5 mm/min, cell load 50 N. The two ends of the patch were fixed with clamps to the dynamometer. The sample was subjected to tensile stress until deformation and break. Values for maximum stress, deformation at maximum stress, and elastic modulus were registered. The obtained results are an average of five measurements (n = 5).

#### 2.2.5. Ex Vivo Adhesion Studies

Patches adhesion force and time were assessed using pig skin samples (from shoulder region), obtained from Large White pigs weighing ~165–175 kg, supplied by Veterinary Service of ASL N. 1 Città di Castello (Perugia, Italy) and used within 12 h from pig death. The ex vivo adhesion force was measured by a dynamometer (Didatronic, Treni, Italy). The patch was attached to support, connected to the dynamometer, using cyanoacrylate glue. A piece of porcine skin tissue was fixed with cyanoacrylate glue on the surface of glass support placed in a thermostatic bath at 32.0 °C ± 0.5. Every patch was cut in portions of 2 cm × 2 cm. The free side of the patch was wetted with 100 μL of SWF and put in contact with the skin sample by applying a light force for 1 min. The force necessary for patch detachment from the skin was measured and expressed as an average of three measurements (n = 3).

#### 2.2.6. Morphology and Thickness

Film morphology and thickness were evaluated by FE-SEM LEO 1525 ZEISS (Carl Zeiss Microscopy, Jena, Germany). The samples were prepared by deposition of the sample on conductive carbon adhesive tape and then metalized with chromium (8 nm) by sputtering.

#### 2.2.7. FT-IR Analysis

Infrared (IR) spectra of the samples were registered by a Shimadzu IR Spirit QATR-S spectrometer (Kyoto, Japan).

#### 2.2.8. Cytotoxicity Assay

HaCaT cell line (300493, CLS Cell Line Service, purchased from I.Z.S.I.E.R. (Istituto Zooprofilattico Sperimentale della Lombardia e dell’Emilia Romagna, Brescia, Italy)) was used to investigate GSE cytotoxicity using the MTT method as previously described [32]. GSE stock solutions (1 mg/mL) were prepared by incubating the compound with complete medium (DMEM) for 1 h at 37 °C. Seven scalar dilutions from 1.875 to 120 µg/mL were tested after 24 h of incubation. OD values were measured spectrophotometrically at 570 nm (Eliza MAT 2000, DRG Instruments GmbH, Marburg, Germany). Each experiment was performed in triplicate three times and cell viability was expressed as a percentage relative, as previously described [33]. A one-way ANOVA test was performed using the Graphpad program (GraphPad Prism 9.2.0.332, GraphPad Software, San Diego, CA, USA).

#### 2.2.9. Scratch Test

GSE effect on wound closure was investigated by using HaCaT cell line by CytoSelect Wound Healing Assay Kit (Cell Biolabs, Inc., San Diego, CA, USA). As previously described [34], cell migration was evaluated after 3, 6, 12, and 24 h of treatment. Four different GSE concentrations were used for the scratch test: 7.5, 15, 30, and 60 µg/mL (based on MTT results). Representative images focused on the center of wound healing were photographed. The influence of compound on wound closure was compared to untreated control (CTR). Three sets of experiments in duplicate were performed.

The total wound field surface area was calculated considering the dimensions of the insert: Total Surface Area = 0.9 mm × 1.8 mm = 1.62 mm^2^. To measure the % closure of the wound field the following equation was used:

(1) $$\%\;\mathrm{closure}=\frac{\mathrm{migration}\;\mathrm{cell}\;\mathrm{surface}\;}{\mathrm{total}\;\mathrm{surface}\;\mathrm{area}}\times 100$$

#### 2.2.10. In Vitro Release Studies

GSE release from the developed patch was studied using vertical Franz diffusion cell USP<1724>, in which the donor chamber was separated from the receptor chamber by a cellulose membrane (Whatman 41, Whatman GmbH, Dassel, Germany). SWF (15 mL) was used as receptor medium maintained at 32 °C by recirculation and magnetically stirred at 600 rpm. The patch (3.14 cm^2^) was placed on top of the cellulose membrane in the donor chamber. Samples were withdrawn at regular time intervals and GSE content was determined by UV analysis (absorbance recorded at λ_max_ = 278 nm, R^2^ = 0.99). All experiments were performed in triplicate, each result represents an average of three measurements and the error was expressed as standard deviation (±SD).

#### 2.2.11. Antibacterial Activity

The antibacterial activity of the samples was evaluated against the strains reported in Table 2. The stored strains were revitalized on BHI Broth and incubated according to the growth conditions shown in Table 2.

The experiments were performed using the agar well diffusion technique, properly modified [32], with a test medium (pH 7.2) prepared according to the recipe previously described (see Section 2.1). After preparation, the test medium was autoclaved. After cooling at 45–48 °C, 1 mL of bacterial suspension was added reaching a final concentration of 1 × 10^5^ CFU/mL. Different inoculated media were used for each bacterial strain. The suspensions were accurately mixed and poured (20 mL) into Petri dishes (90 mm diameter), then let it cool on a horizontal surface. Indeed, 100 µL of products were placed in a hole of 13 mm in diameter, previously made in the center of cooled agar. The plates were incubated according to the growth conditions shown in Table 1. For each bacterial strain, each product was tested and three inoculated agar plates were incubated to verify medium sterility. At the end of the incubation, the presence and the diameter of the inhibition halo were evaluated by a gauge.

## 3. Results and Discussions

### 3.1. Patches Preparation and Characterization

Starting from AG water solution of (5% wt./wt.), a first patch prototype was prepared. The use of 5% wt./wt. was established after preliminary attempts, highlighting that percentages > 5% produce a highly viscous hydrogel hardly to cast. Thus, hydrogels with different AG percentages (1–5% wt./wt.) were prepared. The most promising sample, in terms of consistency and manageability, resulted in the one containing 5% wt. However, the patch obtained from this solution was very sticky and hardly manageable, making difficult the removal from the mold.

According to this, the combination of AG with another polymer was considered useful. With this aim, among the polymers used in the health field, polyvinylpyrrolidone K30 (PVP) was chosen, because largely used in both cosmetic and pharmaceutical formulations, also for topical use. Since it has adhesive properties and easy film-forming ability, is often chosen for the preparation of transdermal patches for the topical delivery of many drugs [10]. Keeping the polymer percentage fixed to 5% wt./wt. in the final hydrogel, different AG/PVP ratios were evaluated and used to prepare patches that were then evaluated in terms of easy removal from the mold with no stickiness and easy handling. The obtained results showed that patch formation occurred using only AG/PVP ratios in which AG prevails (Table 1), in all the other cases the drying did not occur.

### 3.2. Thermal Stability of Patches

**Figure 1:**
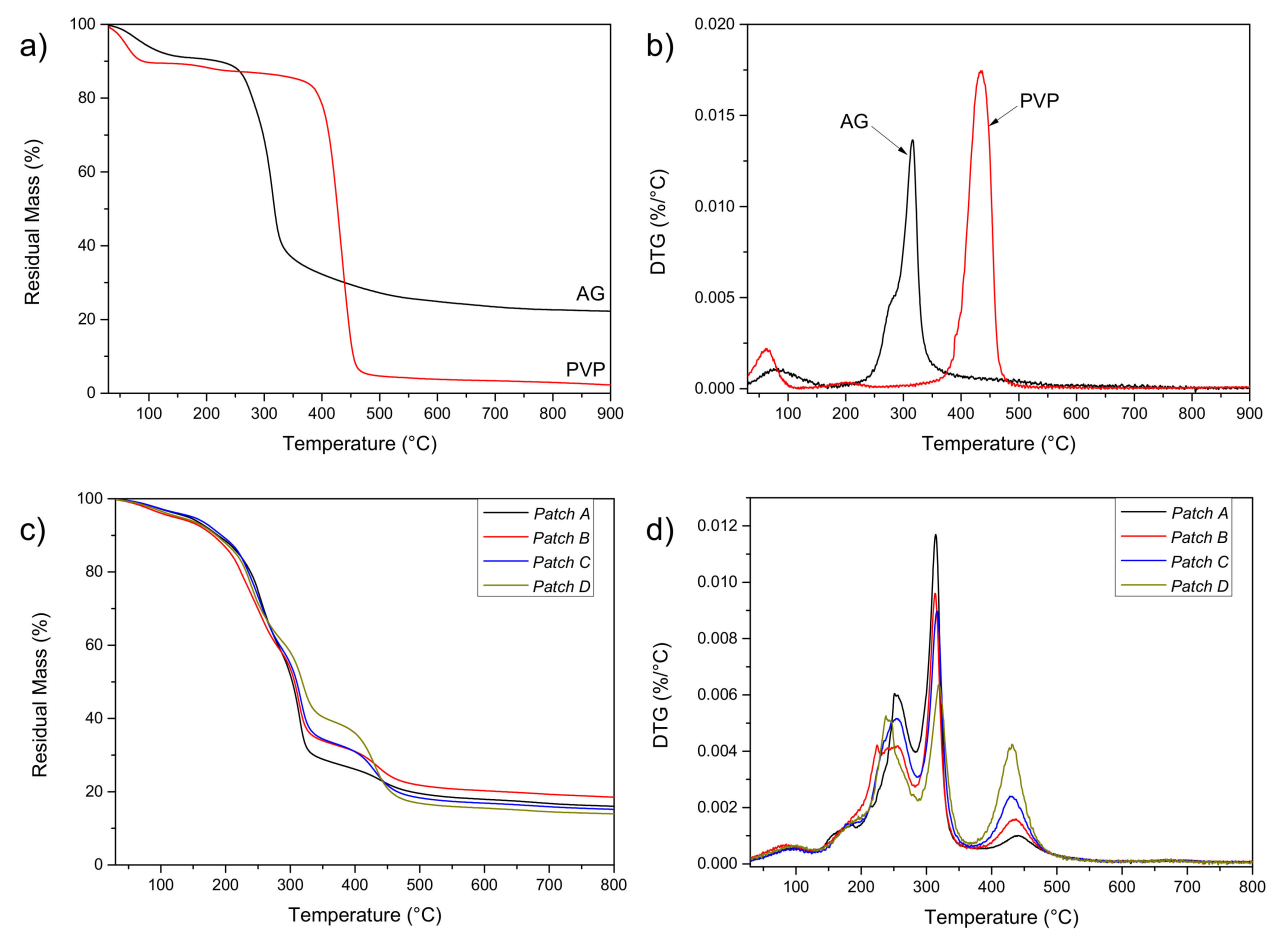
TG (**a**) and DTG (**b**) curves of AG and PVP; TG (**c**) and DTG (**d**) curves of patches A–D.

Firstly, the thermal profile of raw materials (AG and PVP) was performed. TGA profile of the raw AG (Figure 1a) shows, in the range 100–200 °C, a mass loss of ~10% wt. attributable to the water molecules lightly bonded to the saccharide structure by hydrogen bonds. A residual mass of ~25% can be detected at high temperatures (900 °C). A second weight loss is visible in the range of 300–400 °C due to polysaccharide decomposition. PVP (Figure 1a) shows a first weight loss in the temperature range 100–200 °C due to the removal of moisture and low molecular weight oligomers. Then, a second weight loss is detectable in the temperature range of 400–500 °C, due to PVP decomposition.

Analysis of DTG curves (Figure 1b) confirms that the first mass loss for the AG sample is due to the loss of adsorbed and structural water of gum, which takes place between 30 and 150 °C. The polysaccharide decomposition takes place in one step, with an onset temperature of about 250 °C and a maximum degradation temperature of 316 °C (a shoulder is also visible), confirming that AG is a thermally stable material. In the case of PVP, after an initial weight loss due to moisture, the main weight loss approximately occurred at 436 °C due to the depolymerization of the monomers of the main polymer chain.

Patches A–D were also tested by TGA analysis: the obtained results (Figure 1c and Table 2) show, for all the formulations a water loss, at 100 °C, of no more than 4% wt., regardless of the composition. (patch B > patch D > patch A > patch C). These differences are attributable to the different polymer ratios and the different abilities of the AG/PVP to bind water molecules. The DTG curves (Figure 1d) of the four different patches showed the superpositions of profiles for neat AG and PVP components, plus a further peak, between 200 and 250 °C, due to glycerol thermal degradation. The initial weight loss of AG/PVP that began just above room temperature corresponding to water desorption is significantly lower than that observed in both the PVP and AG thermograms. This indicates that a smaller quantity of water is adsorbed in the AG/PVP structure. This is the first signal of the development of specific interactions between AG and PVP polymers, which prevent the establishment of hydrogen bonding between each polymer and water molecule. It is observed that AG polymer is thermally more stable in the blend than in pure AG, as the temperature of maximum degradation rate for AG (T_max_) shifted from 316 °C to 319 °C, this phenomenon can be related to the increase of PVP content: this enhancement in the thermal stability can be attributed to the miscibility of the AG/PVP blend, which is the result of the development of specific interactions between AG and PVP [35].

### 3.3. Mechanical Characterization

As the formulations are supposed to be used on areas of diseased skin, it is important to ensure sufficient flexibility and elasticity because a lack of those properties would result in cracks and fissures disrupting the film upon movements of the patient. Moreover, patches must possess enough resistance to stresses that can be induced during their removal from the packaging, during the application as well as during the period of residence on the skin surface, so information of their stiffness, elongation at the maximum stress should be evaluated. According to this, the following parameters were measured for each formulation (patches A–D):

- maximum tensile strength (σ_max_);
- elongation at maximum strength (ε at σ_max_);
- elastic modulus (E).

**Figure 2:**
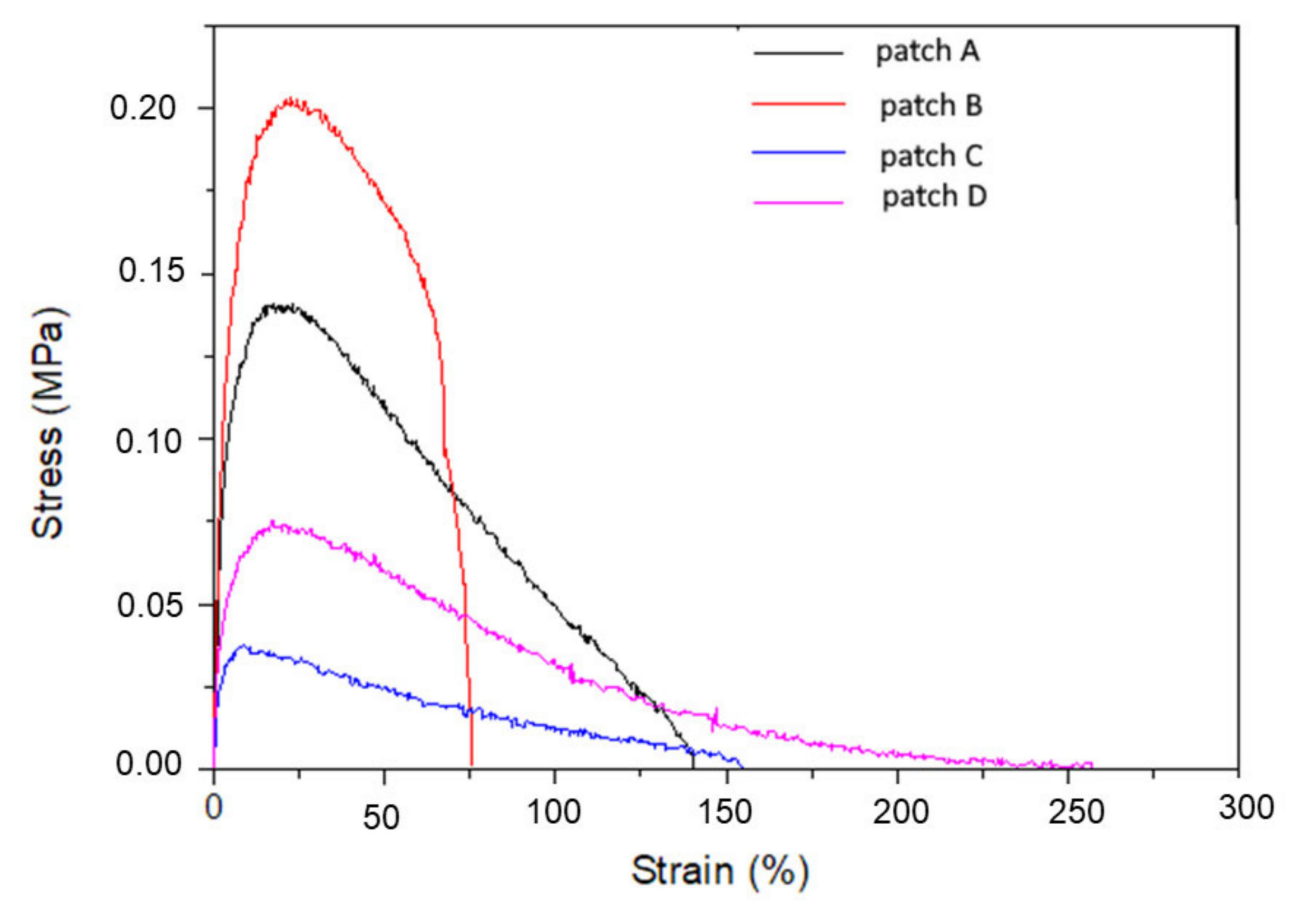
Tensile stress—strain curves for patches A–D.

The experimental stress-strain curves of samples tested after being kept at 50% RH and T = 25 °C for 24 h are reported in Figure 2. If we consider that the same amount of glycerol (3% wt.) was introduced in the blends (regardless of the AG/PVP ratio), no effects due to the plasticizer content can be detected. The considered amount of plasticizer is, however, too low to plasticize the distinct biopolymers (and their blends), but it was intentionally maintained at a limited amount to improve, as pointed out before, handling and limit the stickiness [36] before use. Deformability of patches decreases with increasing amounts of AG (although for patch B a different behavior was observed), while strength was higher in the case of AG:PVP ratios equal to 9.5:0.5 and 9.0:1.0 (Table 3). Because of its unique structure of hydrophobic and hydrophilic segments, it is known that PVP can form complexes with a variety of compounds, that can include one or more of the following types of interactions: hydrogen bonding, polar or hydrophobic attractive forces, stabilization from π-bond overlap. In presence of AG and aqueous media, the mechanical response of the different formulations is hardly understandable: separation of significant different effects (moisture, plasticizer) should be considered, to individuate the role of hydrophilic AG in the blend overall behavior.

Given the designed application, patches with higher tensile strength and acceptable elongation are required: these preliminary studies evidenced that patch D could be the most suitable in terms of elongation capability, nevertheless, the measured tensile strength was limited. Thus, considering this important parameter, both patch A and D can be considered as the best compromise between deformability and strength, so these compositions were considered for the preparation of drug-loaded systems.

### 3.4. Ex Vivo Adhesion Studies

**Figure 3:**
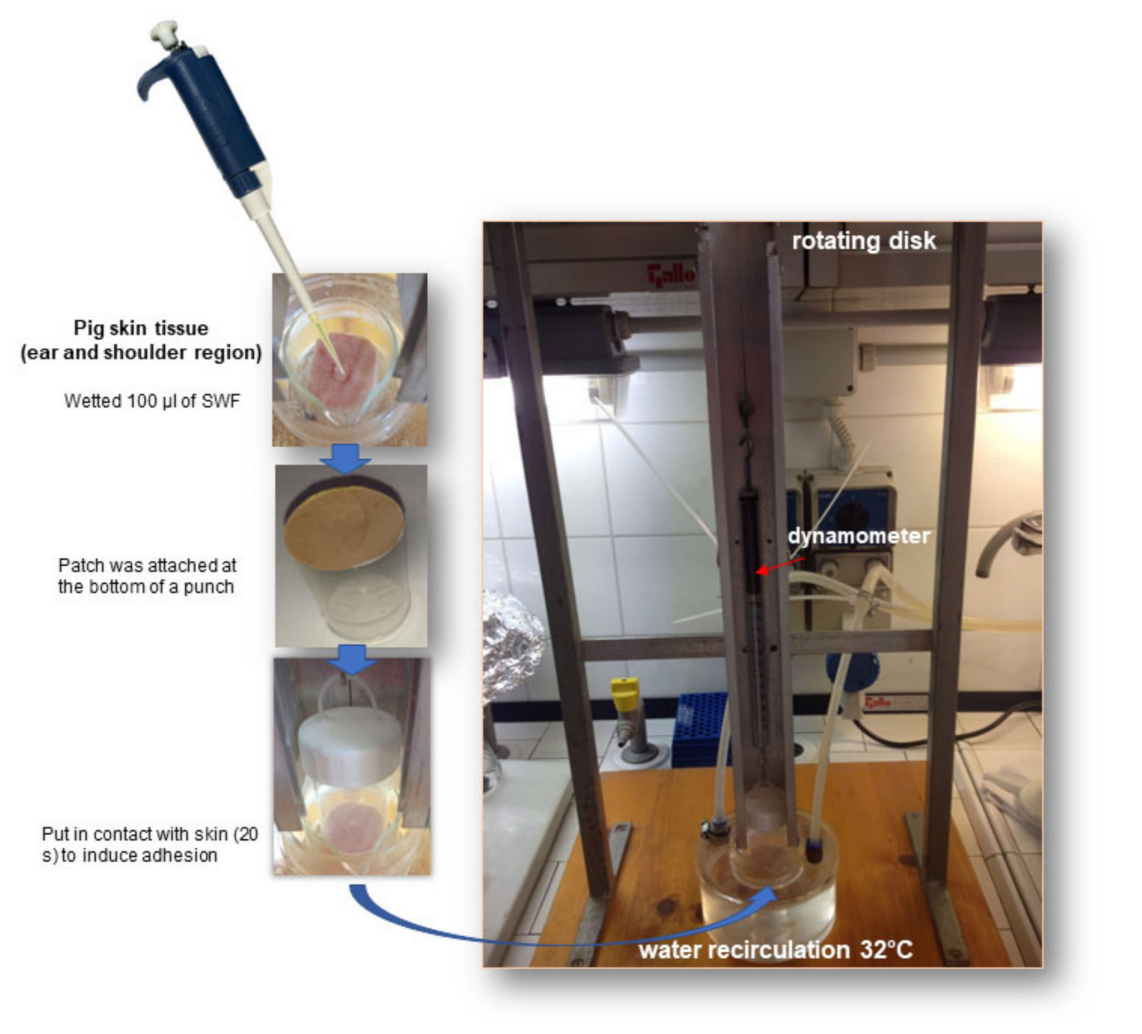
Representation of the experimental setup used to perform ex vivo adhesion studies.

Patch bioadhesion ability was evaluated by ex vivo studies using pig skin samples (2 cm × 2 cm), in which an artificial wound was performed by a scalpel and then filled with 100 μL of SWF simulating the wound exudate (Figure 3). After the contact between the patch and wound (see Section 2.2.5) the force necessary for detachment was measured for patches A–D (Table 4). The experiment was carried out in triplicate and the results were expressed as average ± SD.

The obtained results show that the highest adhesion force was measured for both patches A and D. Natural gums show low bioadhesion capacity has reported in the literature [13] however, the combination with a bioadhesive polymer such as PVP can improve this property.

Considering the composition of patches A and D, it can be hypothesized that PVP chains can interact with the hydrophilic groups of AG (–OH) forming a network structure along with H-bonding between carbonyl groups (C=O) of PVP. The hydrophobic groups (alkyl group for PVP) are exposed to the outer side of the patch and thus available to interact with skin. However, while in the case of patch D the amount of PVP exceeds that of AG, suggesting the availability of many hydrophobic groups to interact with skin, for patch A PVP content is the lowest (~9 times less than AG). It can be hypothesized that the coexistence of the two polymers in this ratio can have a synergistic effect on the final bioadhesion as observed for other authors. To well understand this aspect further studies focused on the evaluation of the inter-polymer interactions are necessary. From these studies patch A and D resulted in the most promising thus chosen for loading.

### 3.5. Loaded Films Preparation and Characterization

Patches A and D were loaded with the GSE as described in Section 2.2.1 GSE is an extract obtained from Vitis vinifera seeds (generally considered food waste and thus thrown out) rich in molecules responsible for the antioxidant, antimicrobial and healing activities [37,38,39], and this reason is useful in a formulation intended for wound treatment. Currently, GSE is formulated as a solution in marketed products purposed for wound treatment. However, these formulations suffer from low residence time that limits their efficacy. Thus, GSE introduction in the developed patch is useful to exploit all the activities.

**Figure 4:**
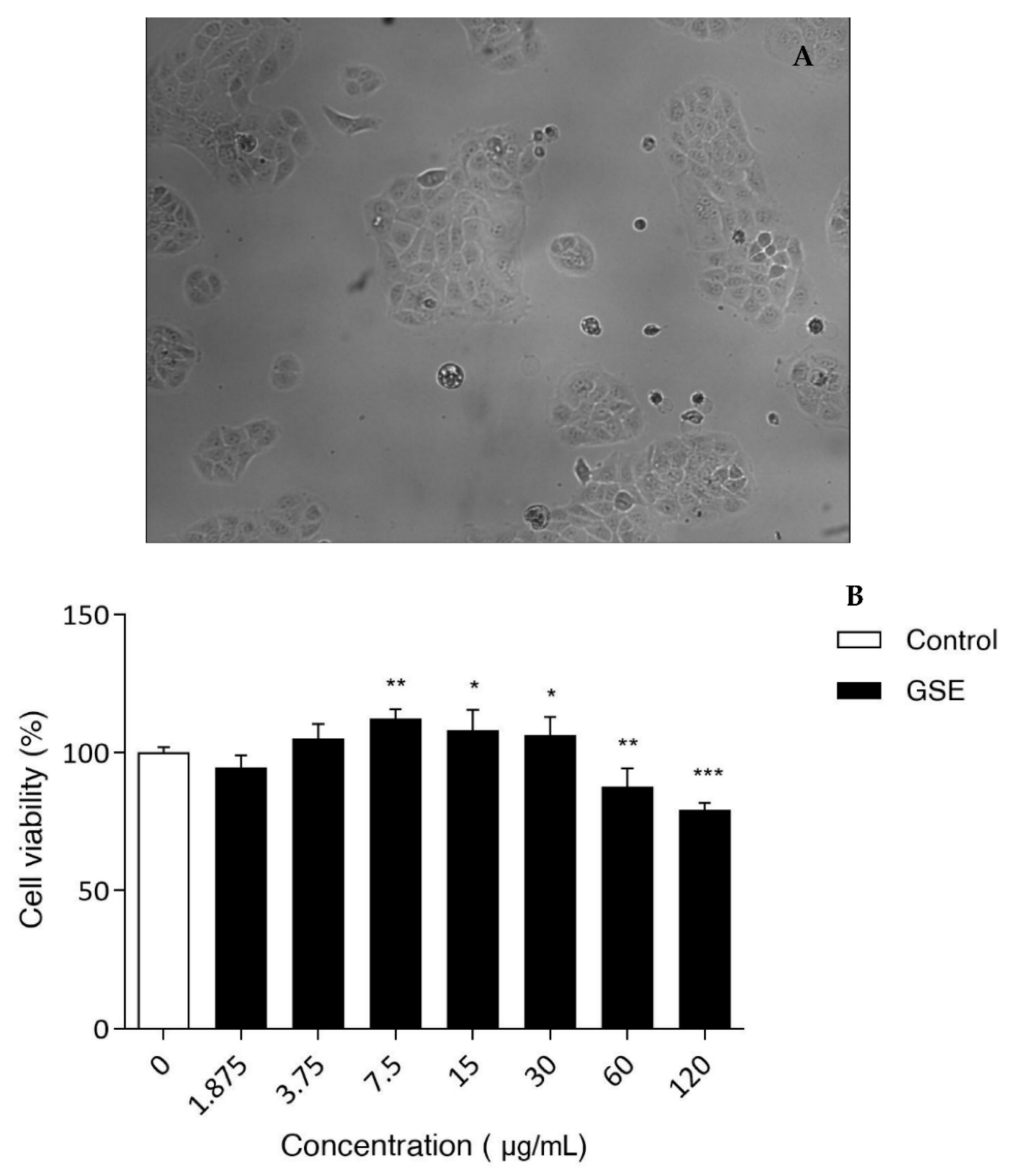
(**A**) HaCaT cells; (**B**) Viability measured in vitro on HaCaT cells for different GSE concentrations. Untreated cells (CTR) were set at 100%. The percentage of viable cells with respect to CTR was reported as the mean standard deviation of three independent experiments, each one conducted in triplicate. * *p* < 0.01, ** *p* < 0.001, and *** *p* < 0.0001, treatments versus CTR (one-way ANOVA test).

As the patch was designed to be applied on the skin, the safety of GSE extract was evaluated as well. Using keratinocytes as a model of epidermidis, in vitro studies were carried out to identify the cytotoxic concentration of GSE. The obtained results showed that this extract is completely safe for HaCaT cells in the range between 1.875–60 µg/mL (Figure 4). Only the highest concentration used (120 µg/mL) showed a notable cytotoxic effect.

Based on MTT results, it is possible to conclude that GSE extract is safe for human keratinocytes at the concentrations assayed and it could be suitable for in vivo application.

#### Scratch Test

**Figure 5:**
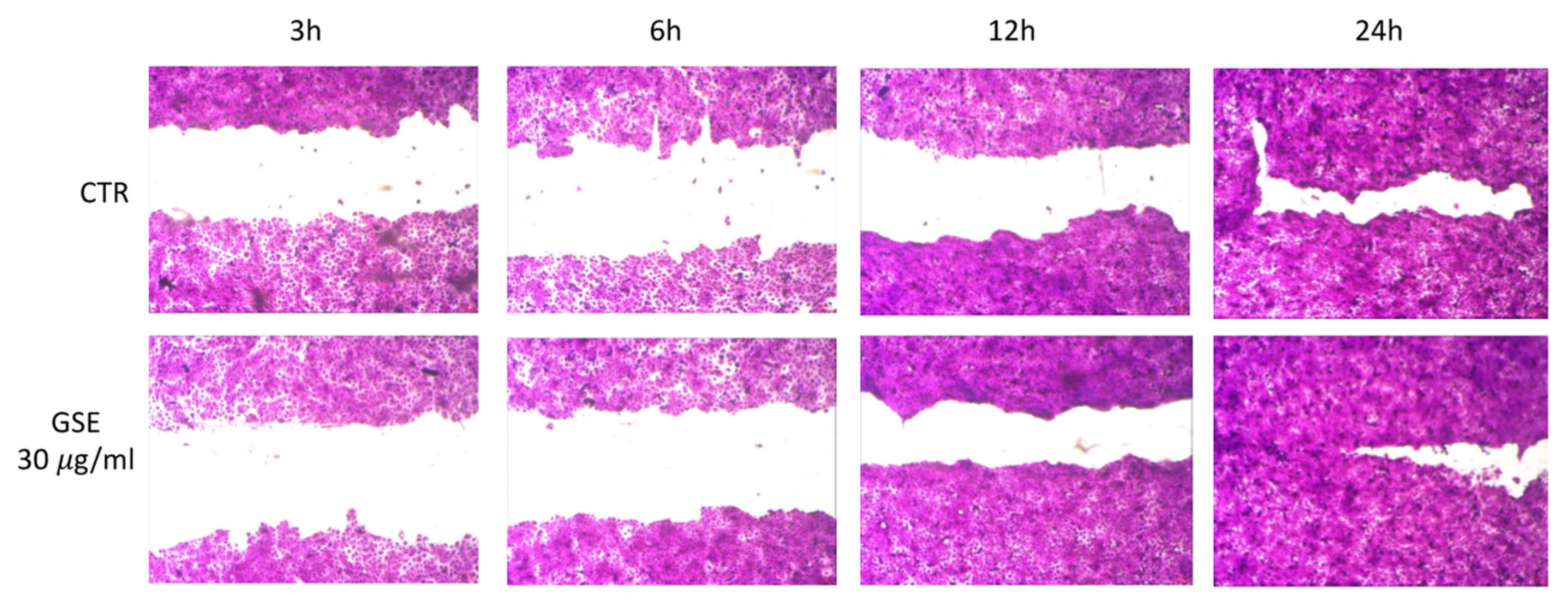
In vitro scratch test on HaCaT cells. Wound closure was observed after 3, 6, 12, and 24 h for untreated cells (CTR) and treated cells with 30 µg/mL of GSE solution.

From the observation of cell viability measured by MTT assay (Figure 4), we decided to investigate a possible GSE ability to stimulate cell growth and wound closure using the following concentrations 7.5, 15, and 30 µg/mL). The latter resulted in the most promising (Figure 5). After 3 and 6 h, no differences were observed between untreated and treated cells. Interesting results were obtained after 12 h of treatment (30 µg/mL) with a decreased area of the wound field (68.4% ± 2.4) compared with CTR (57.1% ± 3.8). The GSE property to stimulate cell growth was confirmed also at the 24 h endpoint of treatment (30 µg/mL) with a wound closure of 96.7% ± 1.9 compared to CTR where the wound field is slightly open (90.5% ± 2.2). This effect could be attributable mainly to proanthocyanidins and tannins contained in GSE as observed from other authors [40]. Based on literature data, one possible reason explaining the reason of the observed effect is represented by GSE’s ability to up-regulate the expression of the vascular endothelial growth factor (VEGF), one of the factors responsible for enhanced wound healing [40,41].

In literature, the benefits of semisolid formulations (cream, ointment) loaded with GSE (2–5% wt./wt.) in wound healing enhancement are reported [21,39,42]. Considering that the prepared patch, after drying in the oven, has a final weight of 10 g and a surface of 162.75 cm^2^, an amount of GSE 0.2 g was dissolved in the starting hydrogel (70 g) to obtain a final patch loading of 2% wt./wt. after drying. The exact hydrogel composition is reported in Table 5.

**Figure 6:**
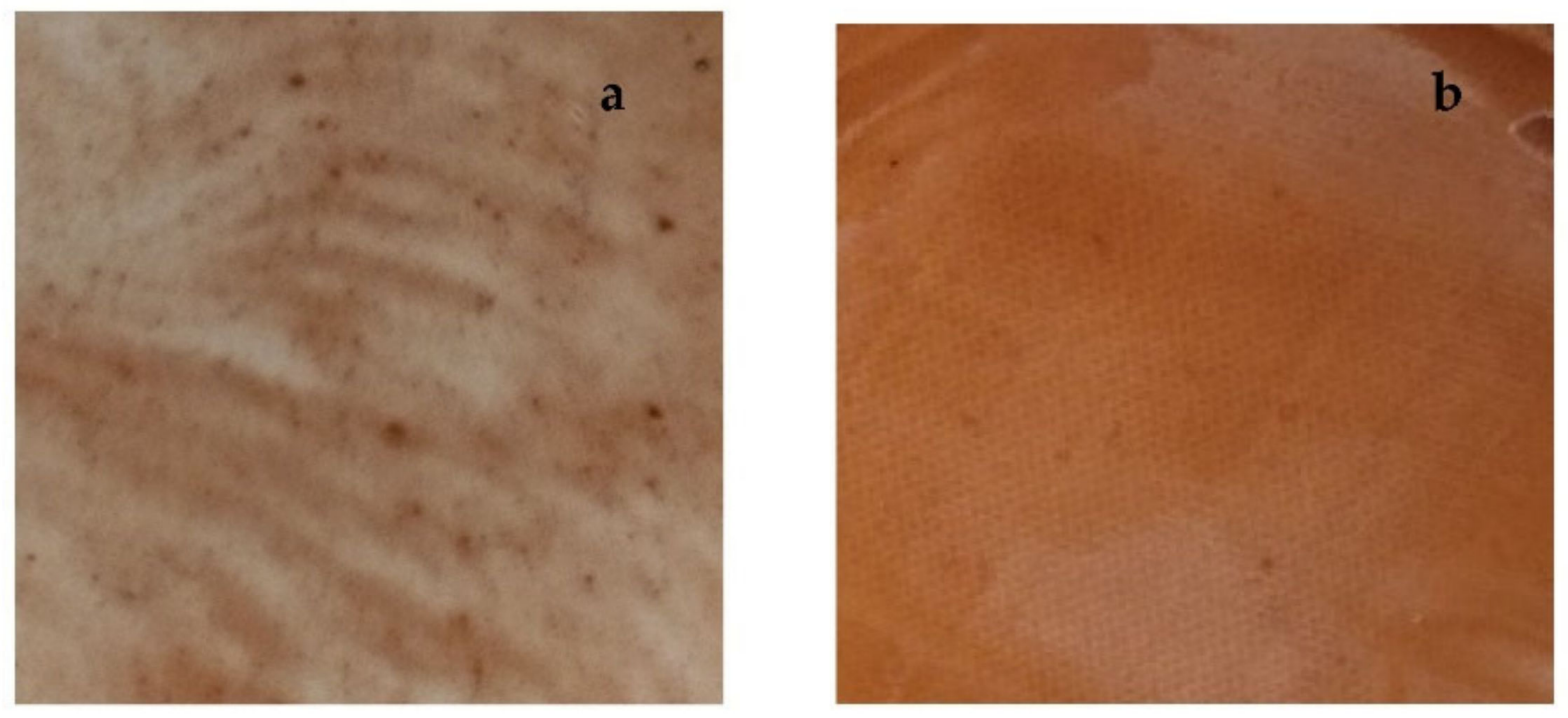
(**a**) Patch A loaded with only GSE; (**b**) Patch A loaded introducing CD in the hydrogel composition.

To obtain a homogenous distribution of GSE in the final patch the introduction of cyclic dextrins (CD) in the final composition was necessary (Table 5). In fact, during the water evaporation in the oven, GSE precipitation occurred with consequent non-homogeneous distribution in the final patch (Figure 6a). Thus, CD introduction in the composition was considered useful as it allows to obtain a final homogeneous patch (Figure 6b). Perhaps, water evaporation represents a driving force inducing GSE inclusion in CD cavities forming complexes soluble in the final xerogel.

**Figure 7:**
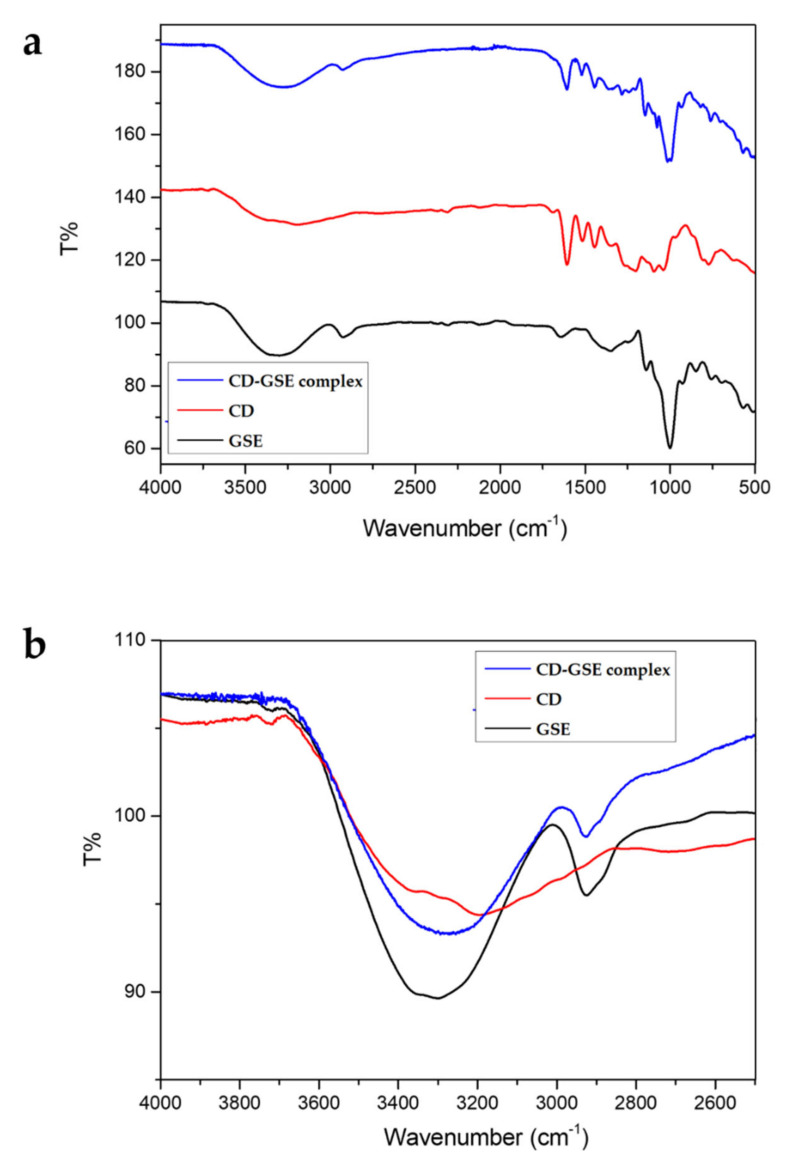
FT-IR spectra of raw materials (**a**). FT-IR spectra of OH stretching (**b**).

To support the hypothesis of GSE-CD complex formation, FT-IR analysis was performed. In particular, CD, GSE, and CD-GSE complex spectra were recorded (Figure 7a). The FT-IR spectra of CD, GSE, and complex CD-GSE exhibited characteristic peaks corresponding to the hydroxyl groups, carboxylic acid groups, C-H and aromatic ring structures at 3300 cm^−1^, 2960–2860 cm^−1^, 1650 cm^−1^, 1455 cm^−1^ 1500–1400 cm^−1^, 1271 cm^−1^, 1321 cm^−1^, and 1200–900 cm^−1^ [43].

Complexation between CD and GSE is highlighted by the prominent shift of the hydroxyl group peak (3300 cm^−1^) of the cyclic dextrin (Figure 7b) due to the formation of hydrogen bonds between the cyclic dextrin and GSE.

Loaded patches A and D were prepared according to the above-described method. However, Patch D showed a fragile behavior, resulting in easily breakable during the removal from the mold. For this reason, it was excluded from the successive studies while patch A resulted most suitable, was further characterized.

**Figure 8:**
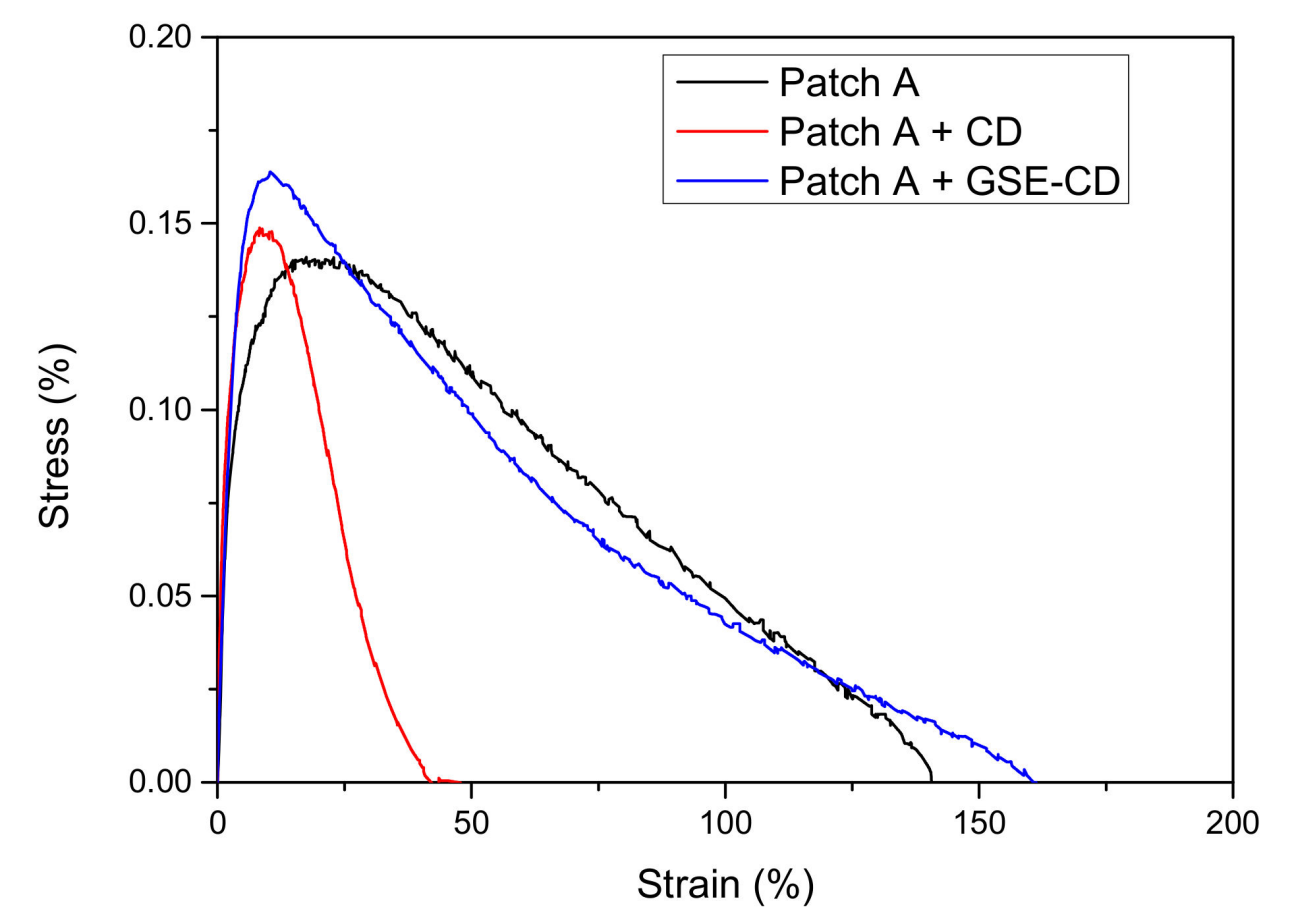
Tensile stress—strain curves for blank, CD and GSE-CD loaded patch A.

Firstly, it was submitted to mechanical characterization to evaluate the influence on this property of CD and GSE-CD introduction in the composition. Results of tensile test for blank patch A, CD, and GSE-CD loaded films are reported in Figure 8 and Table 6.

**Figure 9:**
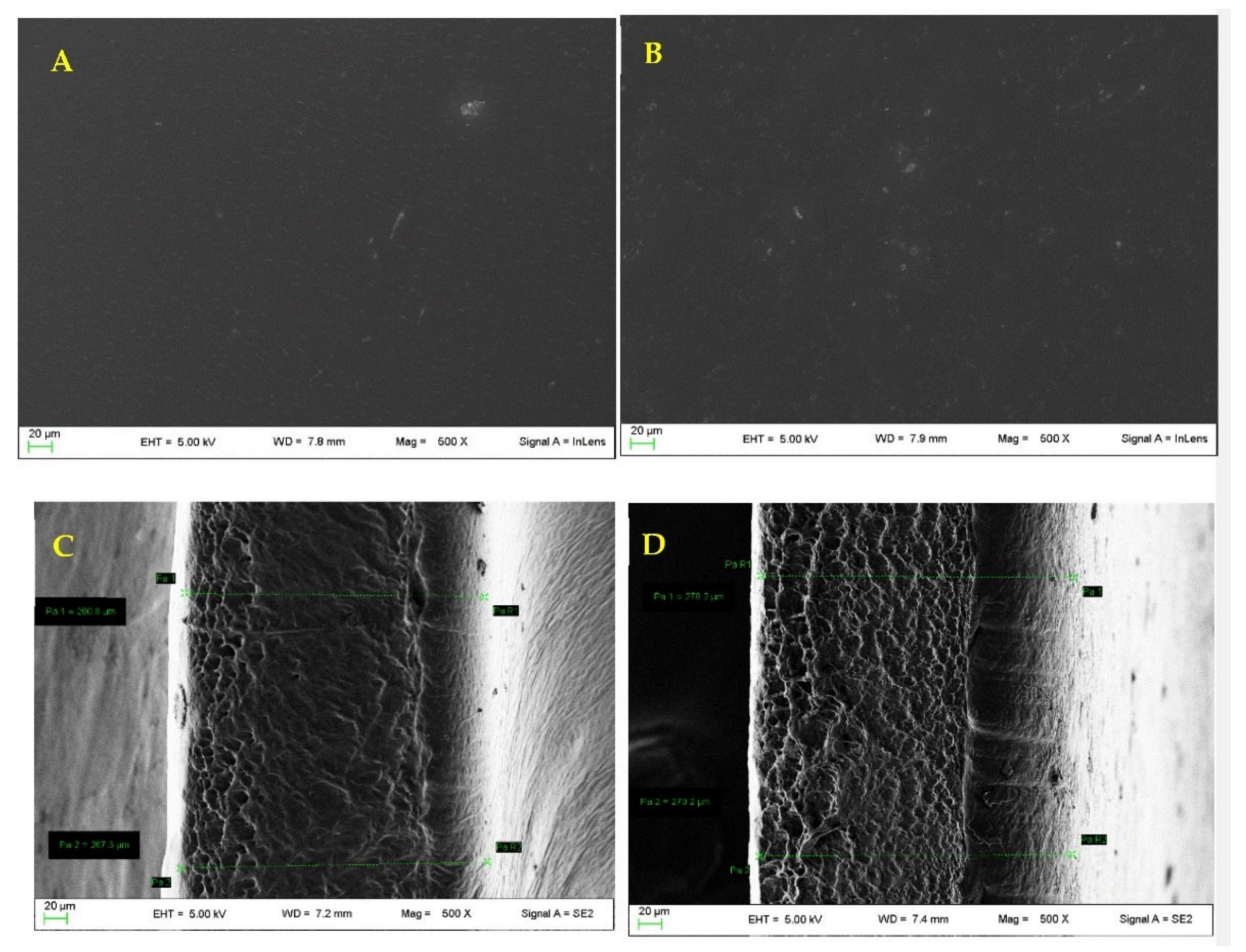
(**A**) Unloaded patch A surface; (**B**) loaded patch A surface; (**C**) unloaded patch A thickness; (**D**) loaded patch A thickness.

The obtained results confirmed that, while Patch A containing only CD is stiffer and less deformable (a reduction in ε both at maximum strength and break was revealed, with a slight increase of the maximum strength to 0.15 ± 0.05 MPa), acting consequently only as a filler able to modify the moisture absorbance of both AG and PVP, Patch A + GSE-CD shows unaltered the tensile strength and the elastic modulus of the blank (Patch A) [44]. The morphological analysis performed (Figure 9) both on empty and loaded patch A showed a smooth surface in the case of the unloaded patch and the presence of small and uniform aggregated in the case of the loaded one. The thickness value increased slightly after loading (from 260 µm ± 0.02 to 270 ± 0.05 µm).

**Figure 10:**
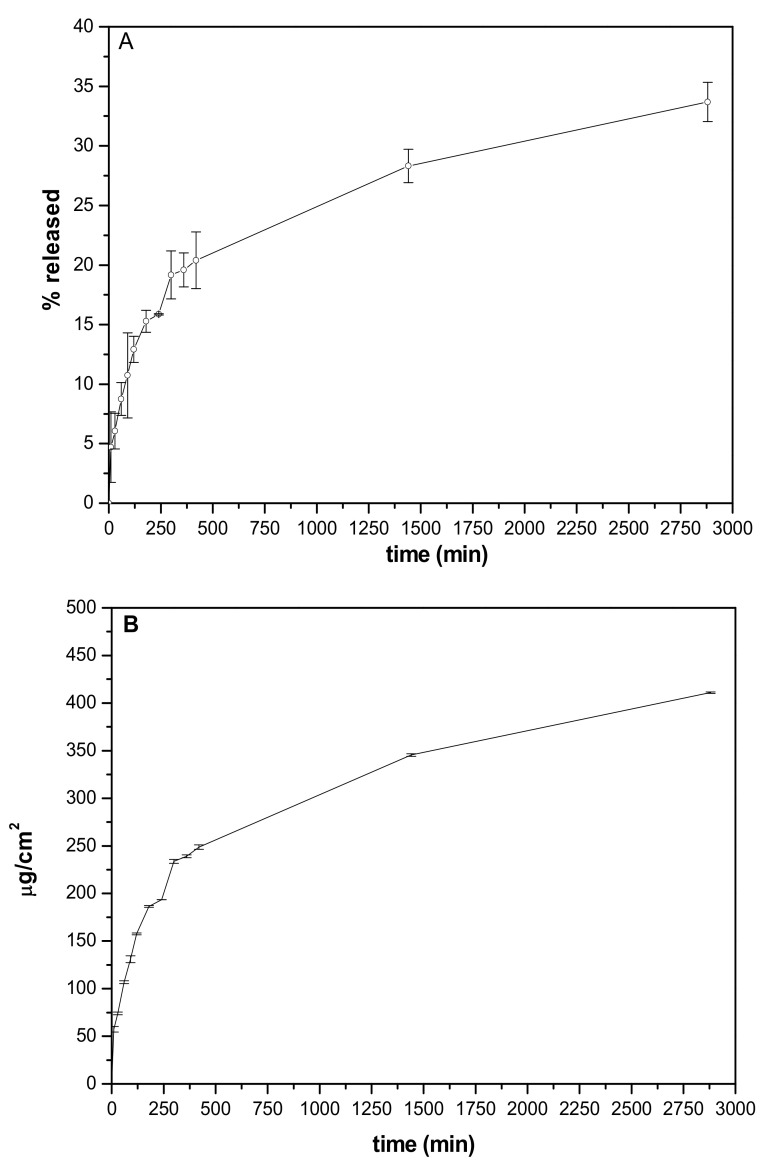
In vitro release profiles were obtained from patch A. (**A**) % released vs. time; (**B**) amount released/cm^2^ (µg/cm^2^) vs. time.

GSE release from the patch was studied using Franz diffusion cell. As observed in Figure 10A GSE was released by a sustained release. The release was ~5.5% after 10 min, ~21% after 7 h reaching 30% at 48th h. These results are justified considering that the experiment is performed in non-sink conditions reproducing the application conditions. The number of fluids available in a wound is very limited (far from skin conditions) suggesting that the in vitro studies performed can be predictive.

The elaboration of release data % by mathematical models (zero-order, first-order, and Higuchi. Zero- and first-order) allowed us to define the kinetic mechanism driving GSE to release from the patch. The obtained results (Table 7) showed the best fitting for Higuchi kinetic suggesting that the diffusion-based mechanism represents the driving force promoting GSE release from the patch.

It was interesting to analyze the results obtained from the elaboration of release data, allowing to measure the amount of GSE released/cm^2^ (Figure 10B). The amount released after 24 h from 1 cm^2^ is efficacious from a different point of view.

Table 8 shows the values of TPC and antioxidant activity (ABTS, DPPH, and FRAP) of empty and loaded films (Patch A and Patch A + GSE-CD, respectively). It can be noted that patch A (empty) showed values near zero to indicate that it did not show the presence of phenolic compounds or other molecules with antioxidant activity, while the loaded patch containing GSE showed higher values for all spectrophotometric determinations, as evidence that the patch containing AG, PVP and CD is provided for antioxidant activity due to GSE presence and that this activity is maintained once introduced in the formulation.

The antimicrobial activity was evaluated as well measuring the inhibition halos for the strains reported in Table 2. The obtained results (Table 9) showed the antimicrobial activity of GSE, supported by literature data [45,46]. The strains that resulted sensitive to GSE are often involved in wound infections suggesting that the projected patch could be useful for this application.

## 4. Conclusions

Acacia gum (AG) was successfully combined with polyvinylpyrrolidone (PVP) to form auto-adhesive patches loaded with grape seed extract useful for wound treatment. This is the first time that grape seed extract is combined with AG, a biocompatible and biodegradable biopolymer, in a formulation for wound treatment.

The most suitable formulation, in terms of mechanical properties (resistance to solicitations, elasticity) and bioadhesion capacity, was obtained using an AG/PVP ratio of 9.5:1 wt./wt. in the starting hydrogel. The presence of the grape seed extract in the final patch is responsible for enhancement. The patch, showing mechanical resistance, offers protection of the damaged area and at the same time demonstrated to control the release of the extract reaching concentrations able to stimulate keratinocytes growth also exerting antioxidant and antimicrobial activities. The advantage of the developed patch resides also in the safety of the active ingredient that allows the treatment of infections resistant to conventional antibiotics. The formulation developed is easy to apply and easily removable (without trauma and pain) respecting the newly formed tissues. The raw materials are easily available and cheap; the production method is scalable and environmentally friendly.

## Figures and Tables

**Table 1:** Compositions of unloaded hydrogel mixtures.

Patch	AG/PVP (w/w)	AG (% wt)	PVP (%)	Glycerol (% wt)	Water (% wt)
A	9.5:0.5	4.75	0.50	3.00	91.75
B	9.0:1.0	4.50	1.00	3.00	91.50
C	8.5:1.5	4.25	1.50	3.00	91.25
D	7.5:2.5	3.75	2.50	3.00	90.75

**Table 2:** Bacterial strains tested and growth conditions.

Bacterial Strains		Growth Conditions
Gram+	*Staphylococcus epidermidis* WDCM 00036	37 °C for 24 ± 2 h
	*Bacillus subtilis* WDCM 00003	30 °C for 24 ± 2 h
	*Staphylococcus aureus* WDCM 00034	37 °C for 24 ± 2 h
	*Streptococcus pyogenes* ATCC 19615	37 °C for 24–48 h
Gram−	*Pseudomonas aeruginosa* WDCM 00025	25 °C for 24–48 h
	*Klebsiella pneumoniae* WDCM 00097	37 °C for 24 ± 2 h
	*Escherichia coli* WDCM 00013	37 °C for 24 ± 2 h
Yeast	*Candida albicans* WDCM 00054	25 °C for 24–72 h

**Table 3:** Tensile parameters for patches A–D.

Patch	σ_max_ (MPa)	ε_at σmax_ (%)	E (MPa)
A	0.13 ± 0.02	23 ± 2	4.00 ± 0.18
B	0.17 ± 0.04	19 ± 5	5.00 ± 1.59
C	0.03 ± 0.01	12 ± 2	2.17 ± 1.15
D	0.05 ± 0.03	19 ± 3	1.67 ± 0.66

**Table 4:** Adhesion forces were measured for empty patches.

Patch	Bioadhesion Force (N) ± SD
A	0.40 ± 0.01
B	0.37 ± 0.06
C	0.30 ± 0.01
D	0.42 ± 0.03

**Table 5:** Loaded hydrogels composition (for 70 g of hydrogel).

Hydrogel	AG (g)	PVP (g)	Glycerol (g)	GSE (g)	CD (g)	Water (g)
A	3.325	0.350	2.100	0.200	0.300	63.725
D	2.625	1.750	2.100	0.200	0.300	63.025

**Table 6:** Tensile parameters for blank, CD and GSE-CD loaded patch A.

Formulations	σ_max_ (MPa)	ε_at σmax_ (%)	E (MPa)
Patch A	0.13 ± 0.02	23.0 ± 2.0	4.0 ± 0.2
Patch A + CD	0.15 ± 0.05	9.0 ± 1.0	6.7 ± 0.2
Patch A + GSE-CD	0.15 ± 0.02	10.0 ± 1.0	4.6 ± 0.6

**Table 7:** Mathematical models of in vitro release data.

M_t_/M_∞_ = kt	M_t_/M_∞_ = kt^0.5^	M_t_/M_∞_ = 1−e^−kt^
Zero-Order Kinetic	Higuchi Kinetic (Release 0–60%)	First Order Kinetic
y = 0.0081x + 11.836 R^2^ = 0.6926	y = 0.5329x + 6.4844 R^2^ = 0.8844	y = −4 × 10^−5^x − 0.0549 R^2^ = 0.7365

**Table 8:** Values obtained for patch having a surface of 1 cm^2^.

Formulations	TPC (mg GAE/cm^2^ Patch)	ABTS (mg TE/cm^2^ Patch)	DPPH (mg TE/cm^2^ Patch)	FRAP (mg TE/cm^2^ Patch)
Patch A	0.05 ± 0.00	0.08 ± 0.00	n.d.	0.02 ± 0.00
Patch A + GSE-CD	1.61 ± 0.03	4.90 ± 0.12	2.55 ± 0.08	0.25 ± 0.00

n.d.—not detectable.

**Table 9:** Inhibition halos measured by the assay of GSE solution at 24th h.

Bacterial Strains		(mm)
Gram+	*S. epidermidis*	27
	*B. subtilis*	21
	*S. aureus*	27
	*S. pyogenes*	18
	*L. monocytogenes*	22
Gram−	*P. aeruginosa*	15
	*K. pneumoniae*	19
	*E. coli*	9
Yeast	*C. albicans*	13

## Data Availability

Not applicable.
